# Signal functions for emergency obstetric care as an intervention for reducing maternal mortality: a survey of public and private health facilities in Lusaka District, Zambia

**DOI:** 10.1186/s12884-017-1451-0

**Published:** 2017-09-06

**Authors:** Tannia Tembo, Gershom Chongwe, Bellington Vwalika, Lungowe Sitali

**Affiliations:** 1 Department of Public Health, School of Medicine, University of Zambia, P.O Box 32379, Lusaka, Zambia; 2 Centre for Infectious Disease Research in Zambia, P.O Box 34681, Lusaka, Zambia; 3 Department of Obstetrics and Gynecology, University Teaching Hospital, P.O Box RW1, Lusaka, Zambia

**Keywords:** Emergency obstetric care, Maternal health, Signal functions, Private, Public

## Abstract

**Background:**

Zambia’s maternal mortality ratio was estimated at 398/100,000 live births in 2014. Successful aversion of deaths is dependent on availability and usability of signal functions for emergency obstetric and neonatal care. Evidence of availability, usability and quality of signal functions in urban settings in Zambia is minimal as previous research has evaluated their distribution in rural settings. This survey evaluated the availability and usability of signal functions in private and public health facilities in Lusaka District of Zambia.

**Methods:**

A descriptive cross sectional study was conducted between November 2014 and February 2015 at 35 public and private health facilities. The Service Availability and Readiness Assessment tool was adapted and administered to overall in-charges, hospital administrators or maternity ward supervisors at health facilities providing maternal and newborn health services. The survey quantified infrastructure, human resources, equipment, essential drugs and supplies and used the UN process indicators to determine availability, accessibility and quality of signal functions. Data on deliveries and complications were collected from registers for periods between June 2013 and May 2014.

**Results:**

Of the 35 (25.7% private and 74.2% public) health facilities assessed, only 22 (62.8%) were staffed 24 h a day, 7 days a week and had provided obstetric care 3 months prior to the survey. Pre-eclampsia/ eclampsia and obstructed labor accounted for most direct complications while postpartum hemorrhage was the leading cause of maternal deaths. Overall, 3 (8.6%) and 5 (14.3%) of the health facilities had provided Basic and Comprehensive EmONC services, respectively. All facilities obtained blood products from the only blood bank at a government referral hospital.

**Conclusion:**

The UN process indicators can be adequately used to monitor progress towards maternal mortality reduction. Lusaka district had an unmet need for BEmONC as health facilities fell below the minimum UN standard. Public health facilities with capacity to perform signal functions should be upgraded to Basic EmONC status. Efforts must focus on enhancing human resource capacity in EmONC and improving infrastructure and supply chain. Obstetric health needs and international trends must drive policy change.

## Background

Maternal mortality is a global health burden and affects women of reproductive age (15–49). It is most acute in developing countries, where complications related to pregnancy and childbirth are among the leading causes of severe disability, mortality and morbidity of women with a report of 358,000 maternal deaths occurring in sub-Saharan Africa [1]. Over time, several promising strategies have been redefined and new interventions adopted to address the problem of maternal mortality. Interventions have focused on reducing the time between onset of a pregnancy complication and delay in decision to seek care, delay in arriving at a health facility or delay in receiving care. Global health experts have identified signal functions for Emergency Obstetric and Newborn Care (EmONC) as the most effective medical intervention for managing direct maternal complications and improving maternal survival [2]. This requires that there are adequate drugs, supplies, equipment, infrastructure, trained staff to competently diagnose and treat complications and equitably distributed health facilities to cater for the needs of populations. Despite evidence of signal functions as an effective medical intervention for managing obstetric complications, maternal mortality remains a challenge in many low and medium income countries including Zambia and many women continue to die due to unpredictable but preventable obstetric complications such as hemorrhage, pre-eclampsia/eclampsia, ruptured uterus, sepsis, and retained placenta, HIV/AIDS and anemia [3].

Zambia has recorded significant declines in maternal mortality ratio (MMR) from 729 per 100,000 live births in 2003 to 398 per 100,000 live births between 2013 and 2014. Despite these reductions, the country was not able to meet its Millennium Development Goal (MDG) 5 target of 162 per 100,000 live births by 2015. Since then, the Ministry of Health (MoH) has redirected efforts to the Sustainable Development Goal (SDG) 3, which aims at reducing the global MMR to less than 70 per 100,000 live births by 2030 [4–6].

Signal functions for EmONC consist of life-saving treatments and procedures including parenteral antibiotics, anticonvulsants and uterotonics, manual removal of placenta, removal of retained products, newborn resuscitation, assisted vaginal delivery, cesarean sections and blood transfusion. They must be available at a health facility 24 h a day, 7 days a week, meet the needs of every 500,000 population and be performed over a designated 3-month period. Health facilities are classified as Basic EmONC (BEmONC) if they have performed seven signal functions (except cesarean section deliveries and blood transfusions) while Comprehensive EmONC (CEmONC) health facilities, usually hospitals, should have performed all signal functions [2].

Signal functions that are medical treatments are more likely to be performed than procedures. A study conducted in 13 developing countries revealed that parenteral antibiotics and uterotonics were more likely to be performed than assisted vaginal deliveries. Equipment, staff, infrastructure, drugs and supplies are important predictors of the level of preparedness for a health facility to manage obstetric emergencies using signal functions [7]. Findings of an assessment in Kwara State, Nigeria showed that a higher proportion of health facilities had provided BEmONC with availability of staff identified as a significant predictor for readiness to provide EmONC. The assessment of 258 public and private health facilities in Benin City revealed that 182 of the health facilities did not meet the BEmONC criteria, while only 4 met the standard for CEmONC. Private health facilities accounted for more health facilities than health facilities in the public sector and were therefore more likely to meet the standard of care for obstetric emergencies [8]. However, findings in China revealed that the number of CEmONC health facilities was adequate and above the recommended minimum of 1 per 500,000 for the population in seven counties while the number of BEmONC health facilities were short of the minimum recommended standard of 4 per 500,000 [9].

Considering that the private sector is increasingly meeting obstetric needs in many countries, private health facilities must also be assessed for obstetric emergency preparedness. There is a dearth of knowledge on the availability and use of signal functions in Zambia as previous assessments undertaken at national level have evaluated the availability and distribution of EmONC in health facilities in rural settings. These findings rarely include data from private health facilities. This lack of evidence therefore underestimates the availability of EmONC particularly in Lusaka where private health facilities provide a significant portion of health care services [2, 10, 11]. This study aimed to evaluate the use of signal functions for EmONC as an intervention for reducing maternal mortality. The survey provides evidence of availability, accessibility, usability and quality of signal functions for EmONC in public and private health facilities in Lusaka district of Zambia.

## Methods

This facility-based cross-sectional survey was aimed at determining availability, accessibility, usability and quality of signal functions for EmONC in public and private health facilities in Lusaka district of Zambia. The district was selected because of its high number of private health facilities, good road network, easy accessibility to referral services and availability of staff to provide obstetric services. Permission to conduct the study was sought from the Zambian Ministry of Health (MoH) while ethical approval was given by the Excellence in Research Ethics and Science Converge Institutional Review Board (ERES Converge IRB).

A list of private and public health facilities was obtained from the Health Professions Council of Zambia (HPCZ). The health care delivery system consisted of 289 hospitals and clinics providing comprehensive clinical care or Anti-Retroviral Therapy (ART), dental, laboratory, male circumcision, ophthalmic, palliative care and physiotherapy services. Two hundred fifty (250) health facilities were listed as ART, dental, industrial nursing homes, laboratory, male circumcision, ophthalmic, palliative or physiotherapy clinics and were therefore excluded from the survey. Letters of request, data collection tools and interview schedules were sent to 39 health facilities to schedule interviews but only 35 health facilities which responded were visited. Access to the health facilities was granted by the Lusaka provincial and district health offices and supervisors at public and private health facilities.

The research team consisted of the first author, a research assistant and two data collectors who were trained for two days and provided with Standard Operating Procedures (SOPs) containing step-by-step instructions on how to extract data from Health Management and Information System (HMIS) registers. Written consent was obtained from in-charges, hospital administrators or staff in maternity wards after which the first author conducted interviews using an adapted Service Awareness and Readiness Assessment (SARA) tool [12]. The tool quantified EmONC services, essential drugs, equipment, infrastructure and staffing while the health facility summary form was used to extract retrospective (June 2013 and May 2014) data on cesarean sections, deliveries, obstetric complications, referrals, maternal and neonatal deaths. Equipment, drugs and supplies were directly observed to determine availability, functionality and validity.

Data were entered into Excel 2013 (Microsoft Office 2013, Version 15.0) while completed questionnaires were scanned into Teleform (Teleform Desktop, Version 10.8–10,844, USA). Verification and cleaning were done by comparing scanned data with source data before committing them to the Teleform database. The Excel file was converted into a Stata dataset using Stata version 11.0 (Stata Corp. Version 11.0, College Station, Texas, USA) for analysis. The study used the UN process indicators to calculate the number of annual expected births, coverage of EmONC per 500,000 population, number of expected direct obstetric complications, met need for EmONC, cesarean section as a proportion of all births and case fatality rate [2]. Facility preparedness to manage obstetric emergencies was determined by the availability of equipment, infrastructure, drugs and supplies, skilled health care providers and their ability to use signal functions for EmONC.

## Results

A total of 35 (25.7% private and 74.3% public) health facilities were assessed. While all the health facilities had provided antenatal care three months prior to the survey, only 22 (25.7% private and 37.1% public) were staffed and operational 24 h a day, seven days a week. A total of 5 (55%) private and 8 (31%) public health facilities had administered antibiotics parenterally for treatment of postpartum sepsis. Overall, 14 (40%) of the public and private health facilities had provided parenteral anticonvulsants for the treatment of preeclampsia/ eclampsia. Of these, 4 (44%) were private and 10 (38%) were public. Parenteral uterotonics for the management of postpartum hemorrhage were performed at all private health facilities while only 11 (42%) of the public health facilities had recorded their use. Manual removal of placenta and retained products were performed more at 11 (42%) and 9 (35%) public health facilities, respectively than they were at private 4 (44%) and 6 (76%) health facilities. Generally, newborn resuscitation and parenteral uterotonics were more likely to be performed at 20 (57%) of public and private health facilities. Even though all health facilities obtained blood products from the only blood bank at one of the two government tertiary hospitals, 7 (78%) private hospitals were more likely to provide blood transfusions as compared to 3 (12%) public health facilities. Eight (88.8%) of the private health facilities also recorded consistent use of assisted vaginal delivery and cesarean section. Despite evidence of obstructed/ prolonged labor being the second attributable cause of direct complications and an indication for emergency surgery, cesarean sections and blood transfusion were the least likely signal functions to be performed at public 3 (12%) health facilities. On the other hand, private health facilities reported performance of cesarean section at 8 (8%) and blood transfusion at 7 (78%) three months prior to the survey (Table 1).

**Table 1:** Health Facility Services and Signal Function Performance

	Private Health Facility Frequency (%)	Public Health Facility Frequency (%)	Overall (%)
	(*n* = 9)	(*n* = 26)	(*n* = 35)
Obstetric and Newborn Services			
Antenatal care	9(100)	26(100)	35(100)
Staffed 24 h/day, 7 days/week	9(100)	13(50)	22(63)
Delivery and newborn care 24 h/day, 7 days/week	9(100)	13(50)	22(63)
Signal Functions in 3 Months			
Parenteral Antibiotics	5(55)	8(31)	13(37)
Parenteral Anticonvulsants	4(44)	10(38)	14(40)
Parenteral Uterotonics	9(100)	11(42)	20(57)
Manual Removal of Placenta	4(44)	11(42)	15(43)
Newborn Resuscitation	8(88)	12(46)	20(57)
Removal of Retained Products	6(67)	9(35)	15(43)
Assisted Vaginal Delivery	8(88)	8(31)	16(46)
Cesarean Section	8(88)	3(12)	11(31)
Blood Transfusion	7(78)	3(12)	10(29)

In this study, 2 (5.7% private and 8.6% public) had potential to become BEmONC health facilities because they had performed six signal functions. At least 3 (5.7% private and 2.9% public) health facilities had performed between seven and eight signal functions, thereby qualifying as BEmONC health facilities. CEmONC signal functions were performed at 2 (5.7%) private and 3 (8.6%) public hospitals. Even though the CEmONC hospitals were adequate for the population of Lusaka, the available BEmONC health facilities fell short of the UN requirement and therefore needed 4.6 (14) times as many health facilities to cater for the BEmONC needs of the Lusaka population. Lusaka district had a population of 2,281,702 and a crude birth rate (CBR) of 5%. Whereas its annual expected births were 114,085, the district recorded 61,182 (53.6%) deliveries in private and public health facilities. Obstetric complications due to direct causes were estimated at 17,113 annually (Table 2).

**Table 2:** Basic EmONC and Comprehensive EmONC Health Facilities

	Private	Public	Total
Population			2,281,702
Number of Expected Deliveries (CBR*Population)			114,085
Total Health Facilities	9	26	35
Total Delivery Facilities	9	13	22
Actual Facility Deliveries	1200	59,982	61,182
Still Births (Fresh and Macerated)	14	1608	1622
Facility Delivery Rate (%)(Actual Deliveries/Estimated Deliveries*100)	1	51	52
Cesarean Sections	446	3685	4131
EmONC Functionality			
Total BEmONC Facilities (6 signal functions)	2	3	5
Total BEmONC Facilities (7 or 8 signal functions) [A]	2	1	3
Total CEmONC Facilities (9 signal functions) [B]	2	3	5
Recommended Number of EmONC Facilities (per 500,000 - [C])			
BEmONC Facilities (Population/500000*A)			18
CEmONC Facilities (Population/500000*B)			5

A total of 7074 obstetric complications were recorded at private (6.2%) and public (93.8%) health facilities. Pre-eclampsia (19.9%) and obstructed/prolonged labor (16.2%) accounted for most obstetric complications while 53.1% of maternal deaths were attributed to postpartum hemorrhage. Approximately 52% of women had delivered in EmONC health facilities and 12% of complications were treated within health facilities. Given the data on deliveries, women with obstetric complications are likely to receive necessary care during obstetric emergencies. Whereas recommendations stipulate 100% treatment for obstetric complications, only 12% of women who needed treatment for obstetric complications received it. Public and private health facilities performed 4577 (4%) elective and emergency cesarean sections. This was below the recommended 5–15%. Recommendations stipulate a maximum acceptable level of 1% case fatality rate. Public and private health facilities documented a case fatality rate of approximately 2%. This rate only included deaths that occurred within the premises of health facilities (Table 3).

**Table 3:** Direct Obstetric Complications and Maternal Deaths in Private and Public Health Facilities

	Private Health Facility	Public Health Facility	Proportion (%)
Direct Maternal Complications			
Complications of abortion	3	290	4.1
Anemia	0	105	1.5
Ectopic pregnancy	3	18	0.3
Obstructed/prolonged labor	50	1079	16
Postpartum hemorrhage	19	473	7
Pre-eclampsia/eclampsia	13	1373	19.6
Puerperal sepsis	0	68	1
Ruptured Uterus	0	25	0.4
Other	353	3202	50.3
Total	441	6528	100
Maternal Deaths in Facilities(By Selected Causes)			
Puerperal sepsis	0	1	3.1
Ruptured uterus	0	4	12.5
Pre-eclampsia/eclampsia	0	8	25
Hemorrhage	0	17	53.1
Other	0	2	6.3
Total	0	32	100

There is a significant relationship between availability of human resources and a health facility’s level of preparedness to manage obstetric complications using signal functions. The use of signal functions was highly dependent on the level of EmONC skill possessed by staff. Private hospitals were better staffed than public health facilities in terms of numbers and specialty as they had all categories of staff to provide support for EmONC. They reported having 66 doctors (general practitioners) and 25 specialists (obstetrician/gynecologists, pediatricians and neonatologist) while public health facilities had 14 and 1, respectively. Obstetrician/ gynecologists were available at public hospitals with CEmONC services only. Obstetric care at public health facilities was likely to be provided by midwives while clinical officer generals (clinical practitioners with 3-year diploma) were available to provide general medical care to outpatients. Medical licentiates (clinical officer general with additional 3-year advanced diploma) performed obstetric surgery and general medical care. As such, there were 245 nurses and 166 midwives at public health facilities as compared to 82 and 51, respectively at private health facilities. Staff working in private health facility were more likely to be trained in EmONC. Overall, there were 44 and 38 health care providers trained in EmONC at private health facilities and public health facilities, respectively. Public health facilities had more (64) clinical officers and medical licentiates than private health facilities which only had four staff in this category. Similarly, there were more (122) lay health workers at public health facilities than at private (3) health facilities. These support staff helped provide non-medical care such as health education during antenatal visits (Table 4).

**Table 4:** Human Resources at Public and Private Health Facilities in Lusaka District

	Public		Private	
	Cadre of Staff	EmONC Trained	Cadre of Staff	EmONC Trained
Doctors (General practitioners)	14	2	66	11
Specialists (Obstetrician/gynecologists, neonatologists, pediatricians, anesthesiologists, surgeons)	1	0	25	5
Clinicians (clinical officer general and medical licentiates)	64	1	4	0
Nursing professionals	245	12	82	9
Midwifery professionals	166	23	51	19
Pharmacists	21	0	13	0
Lay health workers	122	0	3	0
Other (Radiologists, ultra sound technicians, laboratory technologists)	38	0	16	0

The availability of communication, drugs and supplies, equipment and skilled health staff was a key component of a facilities level of preparedness to provide emergency obstetric care. Even though not functional, public health facilities (23.1%) were more likely to have two-way radio for referral communication. Landline telephones were available at 9 (100%) private and 5 (19.2) public health facilities. However, personal or health facility owned cellular phones were used more consistently for emergency referral communication by 34 (97.1%) public and private health facilities. Internet access was most available at private health facilities 8 (88.8%) than at public health facilities 7 (26.9%). Overall, operating theatres were available at 15 (42.9%) of public and private health facilities. Of these, clean delivery kits were available at 22 (62.9%) of health facilities. At least 8 (88.8%) of private health facilities reported availability of Manual Vacuum Aspiration (MVA) and cesarean section packs as compared to public health facilities which reported 18 (69.2%) and 6 (23.1%), respectively. Emergency transport was available at all private health facilities while only 7 (26.9%) of public health facilities reported having an ambulance onsite. Health facilities without ambulances were required to send requests for emergency transport through command post. Only 21 of the 23 health facilities that did not have ambulances onsite had readily available funds for fuel. Generally, health facilities reported availability and validity of essential drugs for emergencies. Resuscitations packs for newborns were available at 8 (88.8%) and 21 (80.7%) private and public health facilities, respectively (Table 5).

**Table 5:** General Supplies and Emergency Transport

	Private	Public	Overall
	(*n* = 9)	(*n* = 26)	(*n* = 35)
Two-Way Radio	0 (0)	6 (23.1)	6 (17.1)
Land Line Telephone	9 (100)	5 (19.2)	14 (40.0)
Facility or Individual Cell Phone	9 (100)	25 (96.2)	34 (97.1)
Functioning Computer	8 (88.8)	15 (55.6)	23 (65.7)
Internet Access	8 (88.8)	7 (26.9)	15 (42.9)
Operating Theatre	8 (88.8)	7 (26.9)	15 (42.9)
Clean Delivery Kits	9 (100)	13 (50)	22 (62.9)
MVA Packs	8 (88.8)	18 (69.2)	26 (74.2)
C/Section Packs	8 (88.8)	6 (23.1)	14 (40.0)
Ambulance at Facility	5 (55.6)	7 (26.9)	12 (34.3)
Ambulance Elsewhere	4 (44.4)	19 (73.1)	23 (65.7)
Fuel for Ambulance	9 (100)	12 (46.2)	21 (60.0)
Neonatal Resuscitation Packs	8 (88.8)	21 (80.7)	29 (82.9)

## Discussion

There is a distinction between theoretical and realistic coverage of EmONC when the UN recommendations are used to classify health facilities as either basic or comprehensive EmONC. The recommendations do not provide guidance on how to distinguish between private and public health facilities. The two types of health facilities should be accounted for separately because private hospitals are designed to provide services for profit and are often well out of the reach of most ordinary Zambians. On the other hand, public health facilities provide services at minimal or no cost and so are within reach of the target population.

Lusaka district was short of the requirements for BEmONC services as stipulated by the UN. These findings correspond well with studies conducted elsewhere in low and high income countries [9, 13–16]. Performance of signal functions was restricted by policy guidelines on obstetric care. For instance, health providers at public health facilities were required to refer pregnant women below 16 and above 30 years old and prime gravidas even if they did not develop complications in labor. At private hospitals, patients with complications were referred to the government tertiary hospital despite being better equipped to handle obstetric complications. Policy change, upgrading of infrastructure and improvement of staff capacity would accelerate the availability of BEmONC services.

There was a shortfall of doctors and specialists such as obstetrician/gynecologists, neonatologists, pediatricians and anesthesiologists at public health facilities. This shortage was likely to affect the quality of care in public health facilities especially because each health care provider was required to attend to more patients than is recommended. The human resources at public health facilities were not adequate to meet the minimum threshold of 23 doctors, nurses and midwives per 10,000 population as established by WHO [17–19]. Many public health facilities reported the availability of staff even though data about their EmONC skills were not readily available. This could be the reason why some facilities were not able to report performance of some signal functions.

Most health facilities were not strictly BEmONC as they had performed between 3 and 6 signal functions 3 months prior to the survey. Private health facilities recorded low numbers of deliveries and thus had minimal obstetric complications to be able to perform the necessary signal functions. The strict use of process indicators to categorize health facilities as recommended by the UN does not reflect the capacity of a health facility to perform EmONC functions [20–22]. In addition, the inconsistency in providing EmONC services caused health facilities to vacillate in status between BEmONC and non BEmONC but does not necessarily reflect compromised quality of emergency services. All basic and comprehensive EmONC health facilities were located within 10 km of the district health office and were within reach for the population in Lusaka. This, coupled with a considerably good road network and reliable public transportation system, made it easier for patients to access basic or comprehensive EmONC services when in need.

Health policies govern how health systems should provide obstetric and newborn care, including which category of health professionals can be trained to provide emergency obstetric services. For instance, magnesium sulfate is recommended for treating women with convulsions due to pre-eclampsia/eclampsia but, its distribution and use in public health facilities was restricted due to the limited number of staff adequately trained in its use [23]. The inability of health facilities to manage women with hypertensive disorders was mirrored in the high numbers of women referred to government tertiary hospital for further management. Considering that pre-eclampsia and eclampsia were the highest cause of direct complications, these data should provide renewed attention towards redefining policy and scaling up the use of anticonvulsants.

Even though private health facilities were better equipped with infrastructure, equipment, drugs and staff to provide EmONC than public health facilities, they handled lower numbers of deliveries and consequently fewer complications but still referred women with obstetric complications to the only tertiary hospital in the district [24].

Although this research was carefully prepared, its results are affected by limitations and shortcomings such as missing registers and inaccurate or incomplete data. At the only tertiary hospital, complications of abortion were documented in registers in the gynecological ward and these data were not collected. This study was not able to determine the geographical distribution of EmONC health facilities due to the non-availability of GIS software to analyze geographical data [25]. Data from private health facilities may not be a generalization of EmONC services provided as some private health facilities were not responsive. Future studies need to be conducted in other urban districts with similar socio-economic characteristics as Lusaka to determine availability, usability and quality of signal functions for EmONC.

## Conclusions

The UN process indicators can be adequately used to monitor progress towards maternal mortality reduction. Lusaka district had an unmet need for BEmONC as health facilities fell below the minimum UN standard. Public health facilities that provide antenatal and postnatal services and are not staffed 24 h a day, seven days a week should be provided with infrastructure for delivery services and upgraded to be able to provide BEmONC services. Health facilities that have capacity to perform at least six signal functions should also be upgraded to BEmONC status. This will reduce the patient load on the referral hospitals within the district and enable health care providers focus on improving the quality of CEmONC. Further, the Ministry of Health must focus on enhancing human resource capacity in EmONC and improving infrastructure and supply chain. Obstetric health needs and international trends must drive policy change. Further, staff audits must be conducted, new staff recruited and evenly distributed. Authorities should consider decentralizing blood bank hubs to make blood products more accessible.

## Abbreviations

AMSTL: Active Management of Third Stage of Labor
BEmONC: Basic Emergency Obstetric and Care
CBR: Crude Birth Rate
CCT: Controlled Cord Traction
CEmONC: Comprehensive Emergency Obstetric and Care
EmONC: Emergency Obstetric and Care
ERES Converge IRB: Excellence in Research Ethics and Science Converge Institutional Review Board
GIS: Geographical Information System
HMIS: Health Management Information System
HPCZ: Health Professions Council of Zambia
MDG: Millennium Development Goals
MoH: Ministry of Health
MVA: Manual Vacuum Aspiration
SARA: Service Availability and Readiness Awareness
SDG: Sustainable Development Goals
SOP: Standard Operating Procedures
UN: United Nations
WHO: World Health Organization
